# Microtubule disrupting agent‐mediated inhibition of cancer cell growth is associated with blockade of autophagic flux and simultaneous induction of apoptosis

**DOI:** 10.1111/cpr.12749

**Published:** 2020-03-13

**Authors:** Mohammad Hasanain, Rohit Sahai, Praveen Pandey, Mayank Maheshwari, Kuldeep Choyal, Deepa Gandhi, Akhilesh Singh, Kavita Singh, Kalyan Mitra, Dipak Datta, Jayanta Sarkar

**Affiliations:** ^1^ Biochemistry Division CSIR‐Central Drug Research Institute Lucknow Uttar Pradesh India; ^2^ Academy of Scientific and Innovative Research Ghaziabad Uttar Pradesh India; ^3^ Electron Microscopy Unit CSIR‐Central Drug Research Institute Lucknow Uttar Pradesh India

**Keywords:** apoptosis, autophagy, cancer, microtubule, TN‐16

## Abstract

**Objectives:**

Given that autophagy inhibition is a feasible way to enhance sensitivity of cancer cells towards chemotherapeutic agents, identifying potent autophagy inhibitor has obvious clinical relevance. Here, we investigated ability of TN‐16, a microtubule disrupting agent, on modulation of autophagic flux and its significance in promoting in vitro and in vivo cancer cell death.

**Materials and methods:**

The effect of TN‐16 on cancer cell proliferation, cell division, autophagic process and apoptotic signalling was assessed by various biochemical (Western blot and SRB assay), morphological (TEM, SEM, confocal microscopy) and flowcytometric assays. In vivo anti‐tumour efficacy of TN‐16 was investigated in syngeneic mouse model of breast cancer.

**Results:**

TN‐16 inhibited cancer cell proliferation by impairing late‐stage autophagy and induction of apoptosis. Inhibition of autophagic flux was demonstrated by accumulation of autophagy‐specific substrate p62 and lack of additional LC3‐II turnover in the presence of lysosomotropic agent. The effect was validated by confocal micrographs showing diminished autophagosome‐lysosome fusion. Further studies revealed that TN‐16–mediated inhibition of autophagic flux promotes apoptotic cell death. Consistent with in vitro data, results of our in vivo study revealed that TN‐16–mediated tumour growth suppression is associated with blockade of autophagic flux and enhanced apoptosis.

**Conclusions:**

Our data signify that TN‐16 is a potent autophagy flux inhibitor and might be suitable for (pre‐) clinical use as standard inhibitor of autophagy with anti‐cancer activity.

## 1 INTRODUCTION

Autophagy is an evolutionarily conserved process of salvaging cellular constituents. It serves as an alternative mechanism of energy production during metabolic stress. Autophagy is a genetically regulated homeostatic control mechanism for maintaining quality of intracellular protein and organelle. The relevance of autophagy in pathophysiology of human diseases is becoming increasingly evident in recent years. Defects in autophagy have been linked with various diseases such as Crohn's disease,^1^ Paget disease of bone,^2^ asthma,^3^ cancer^4^ and several other neurodegenerative,^5^ cardiovascular^6^ and autoimmune disorders.^7^ Many of these diseases have been reproduced in animal models through systemic or tissue‐specific alterations of autophagy regulatory genes.^8^ With the growing evidences on fundamental roles of autophagy in pathogenesis of human diseases, continuous efforts are being made in therapeutic targeting of this cellular process for various human health problems. The rationale of inhibiting autophagy in cancer therapy emanated from the observations of frequently enhanced level of autophagy in cancer cells with oncogenic mutations.^9^ In this endeavour, pharmacological inhibition of autophagy had shown some success in combination with other standard therapies to enhance chemosensitivity of solid tumours.^10^

Direct inhibition of autophagy, by chemical inhibitors, is usually achieved by targeting either at early phase of phagophore formation (eg, 3‐MA, LY294002, Wortmannin) or at late phase of autophagolysosomal proteolysis (eg, Bafilomycin A1, CQ, HCQ). Autophagosomes, formed in cytosol, move along the microtubule track to transport cellular cargo towards lysosomes at perinuclear region, and disruption of tubulin network results in suppression of autophagic process.^11^ By targeting this essential step of autophagic process, microtubule destabilizing agents indirectly inhibit autophagy by preventing autophagosome‐lysosome fusion. Supported with the known pharmacologic and toxicologic profiles, chloroquine (CQ) and hydroxychloroquine (HCQ) have been commonly used in various clinical trials to inhibit autophagy for management of multiple malignancies. However, prolonged use of CQ is associated with various side effects among which retinal toxicity is the most important complication in patients.^12^ On the other hand, relatively safer HCQ has been linked with various potential drawbacks such as higher medication dose to achieve sufficient autophagy inhibition, prolong retention in the system after completion of therapy and various other side effects including retinopathy and indigestion.^13^ Thus, there is obvious room for other potent autophagy inhibitors, with better efficacy, for clinical use.

3‐(l‐anilinoethylidene)‐5‐benzylpyrrolidine‐2,4‐dione (TN‐16) is a tenuazonic acid derivative which was first synthesized in 1983 as novel microtubule (MT) disrupting agent with anti‐tumour activity.^14^ TN‐16 was shown to inhibit tubulin polymerization by binding at colchicine‐sensitive site of MTs.^14^ Subsequently, it was shown to block formation of intra‐chain cross‐linking upon reacting tubulin with N,N'‐ethylene‐bis (iodoacetamide), and the outcome was comparable with the effects of colchicine, podophyllotoxin and nocodazole when tested in identical reaction condition.^15^ Despite being able to interact with same binding site, the structure of TN‐16 is distinct from colchicine and rather showed resemblance with standard actin polymerization inhibitor cytochalasin B.^16^ TN‐16 was also shown to induce cell cycle arrest at M phase^17^ and inhibit cytotoxic effect of CTL and NK cells.^16^

In the present study, we investigated effect of TN‐16 on autophagic process. We found that TN16 is a potent inhibitor of classical autophagy pathway which prevents fusion between autophagosome and lysosome. We have also revealed that diminished autophagic flux in TN‐16–treated cells is associated with induction of apoptosis and these two mechanisms influence each other.

## 2 MATERIALS AND METHODS

### 2.1 Reagents and antibodies

TN‐16 (T7580), chloroquine (C6628), rapamycin (R0395) and bafilomycin A1 (B1793) were purchased from Sigma‐Aldrich. Anti‐LC3B antibody (L7543), anti‐p62/SQSTM1 (P0067) and peroxidase‐conjugated anti‐mouse (A9044) and anti‐rabbit (A0545) antibodies were purchased from Sigma‐Aldrich. Anti‐GAPDH (ABM22C5) antibody was obtained from Imgenex (ABGENEX Pvt. Ltd). Anti‐PARP (9532), anti‐caspase‐3 (9662), anti‐caspase‐9 (9502), anti‐Atg7 (8558), anti‐Atg5 (9980), anti‐beclin‐1 (3495), anti‐bax (5023), anti‐bak (12105) and anti‐cyclin B1 (12231) were procured from Cell Signaling Technology.

### 2.2 Cell culture

The mammary carcinoma cell lines of human (MCF‐7 and MDA‐MB‐231) and mouse (4T1) origin were obtained from American Type Culture Collection (ATCC). Isogenic engineered cells derived from human colon HCT‐116 cancer cells and their parental cell lines were kind gift from Prof. Bert Vogelstein (Johns Hopkins University). MCF‐7, MDA‐MB‐231 and 4T1 cells were grown in RPMI‐1640 medium (Gibco BRL) while HCT‐116 and its isogenic cells were maintained in McCoys's 5A (Sigma‐Aldrich). All media were supplemented with 10% foetal bovine serum (HiMedia Laboratories) and antibiotic‐antimycotic solution (Sigma‐Aldrich). Cells were grown at 37°C in humidified atmosphere containing 5% CO_2_.

### 2.3 Cell proliferation assay

Proliferation of cells, before and after incubation with TN‐16, was determined by sulforhodamine B (SRB) assay as previously described.^18^ Briefly, cells were seeded in 96‐well plates at a density of 10^4^ cells/well and grown overnight in CO_2_ incubator. The following day, TN‐16 was added at various concentrations and cells were incubated further for 48 hours. Viable cells were then fixed with 50% trichloroacetic acid (TCA) and stained with SRB for 30 minutes. Thereafter, excess dye was collected, and after brief washing, protein bound dye was dissolved in 10 mmol/L Tris base solution for colorimetric measurement at 510 nm.

### 2.4 Cell cycle analysis

Effect of TN‐16 on cell cycle distribution was determined by flowcytometric analysis, as described earlier.^19^ Cells were grown overnight in 6‐well culture plate and treated with TN‐16 at different concentrations for 24 hours. The cells were fixed with ice‐cold 70% ethanol (v/v), stained with propidium iodide (PI) solution containing TritonX100 (5%) and RNase (1 mg/mL) for 30 minutes at 37°C in dark and analysed on FACSCalibur™ flow cytometer (BD Biosciences) with inbuilt software.

### 2.5 Immunoblot analysis

Protein extracts from cells, treated with vehicle or TN‐16, were prepared by lysing on ice in a lysis buffer consisting 50 mmol/L Tris (pH 7.4), 150 mmol/L NaCl, 1 mmol/L EDTA, 1 mmol/L EGTA, 1% Triton X100, and 0.5% Tween‐20 and supplemented with protease and phosphatase inhibitor cocktail (Sigma‐Aldrich). T‐PER reagent (Thermo) was used for lysis of tumour tissue samples. The protein concentrations in cell and tissue samples were determined using the bicinchoninic acid (BCA) protein assay kit (Thermo). Equal amount of proteins were electrophoresed on SDS‐PAGE and transferred onto PVDF membranes. Membranes were then blocked with 5% non‐fat milk in 0.1% Tween‐20 in TBS for 1 hour and subsequently incubated with respective primary antibodies as indicated in the figures. Immune complexes were visualized in a Chemidoc XRS + Imaging system (Bio‐Rad) after incubation with appropriate secondary antibodies (horseradish peroxidase conjugated) and subsequent addition of enhanced chemiluminescence (ECL) solution.

### 2.6 Immunocytochemistry

Cells, after growing overnight on coverslips to required confluence, were treated with TN‐16 for indicated time periods and fixed with 4% paraformaldehyde. Cells were subsequently permeabilized (0.5% Triton X100 in PBS), blocked with 2% BSA in PBS and incubated overnight with primary antibodies against specific target proteins at 4°C. Following primary antibodies were used at indicated dilutions for probing target proteins—anti‐LC3A/B antibody (Cell Signaling Technology, 9091; 1:250), anti‐LAMP‐2 antibody (Santa Cruz Biotechnology, sc‐18822; 1:250) and anti‐β‐tubulin (Thermo Fisher Scientific, 480011; 1:250). After 3 × 5 minutes washes in PBS, cells were incubated with corresponding (anti‐mouse Alexa Fluor™ 488, A10468; anti‐mouse Alexa Fluor™ 594, A11032 or anti‐rabbit Alexa Fluor™ 594, A11037) fluorescence‐conjugated secondary antibody (Invitrogen Corp) @1:250 dilutions for 1 hour at room temperature (RT). Following three washes, coverslips were mounted on glass slides using ProLong Gold Antifade reagent (Invitrogen Corp) containing DAPI. Samples were subsequently examined using appropriate excitation and emission filters under LSM510 META confocal microscope (Carl Zeiss) and a Plan Apochromat 63 × 1.4 NA Oil DIC objective lens.

### 2.7 Plasmids and RNA interference

Recombinant plasmid for retroviral expression of GFP‐tagged LC3 (pBABEpuro GFP‐LC3; Addgene plasmid number 22405) was created by Dr Jayanta Debnath.^20^ Another retroviral vector expressing GFP‐LC3‐RFP‐LC3ΔG fluorescence probe (pMRX‐IP‐GFP‐LC3‐RFP‐LC3ΔG; Addgene plasmid number 84572) was constructed by Dr Noboru Mizushima.^21^ Both plasmids were obtained from Addgene. Generation of retrovirus and transduction of experimental cell lines were performed following standard protocol.

Chemically synthesized shRNA sequences targeting Atg7,^20^, ^22^ Bak^23^ and scrambled control (http://www.hollingscancercenter.org/research/shared-resources/shRNA/Vectors.pdf) were procured from Integrated DNA Technologies, Inc. The sequences were annealed and sub‐cloned into the AgeI and EcoRI sites of the pLKO.1 puro vector. Standard protocol was followed for generation of lentivirus and transduction of cells.

Cells, transduced with retro/lentiviruses encoding desired genes/shRNAs, were cultured in growth media as mentioned above. Puromycin was added into the media after 48 hours of transduction. After maintaining the cells for ~10 days in puromycin‐containing media, viable stable clones were propagated and used for experiments.

### 2.8 Scanning electron microscopy

The cells were cultured on glass coverslips in a 6‐well culture plate before incubation with vehicle or TN 16. Cells were then fixed overnight with 2.5% glutaraldehyde in 0.1 mol/L phosphate buffer (pH 7.4) at 4°C and thereafter subjected to osmication. Post‐washing, the samples were dehydrated using ascending grades of ethanol followed by critical point drying (CPD). The samples were then sputter coated with ~15 nm thickness of Au:Pd with a Polaron E5000 sputter coater and imaged at 20 kV in FEI Quanta 250 scanning electron microscope with SE detector. About 200 cells from two stubs for each sample were analysed.

### 2.9 Transmission electron microscopy (TEM)

The study was performed as described previously.^18^, ^24^ Vehicle control or TN16‐treated cells were fixed overnight at 4°C using 2.5% glutaraldehyde in 0.1 mol/L phosphate buffer (pH 7.4) followed by osmication and encapsulation in agarose by centrifugation. Approximately 1 mm^3^ pieces of pellet were cut and subjected to dehydration in ascending series of ethanol and acetone, before finally being embedded into Spurr resin. Sixty to eighty nanometre thick sections were obtained Leica EM UC7 microtome which were collected over 200 mesh copper grids. The sections were double‐stained with uranyl acetate and lead citrate and air‐dried before viewing under JEOL JEM 1400 transmission electron microscope at 80kV using Gatan Orius SC200B CCD camera.

### 2.10 LysoTracker staining

For fluorescence microscopic analysis, MCF‐7 cells were grown overnight on coverslips in 6‐well plates at 37°C and exposed to indicated chemicals for additional 24 hours. During the final 30 minutes of incubation, 50 nmol/L LysoTracker Red DND‐99 (Thermo Fisher, Cat# L7528) was added to the medium. Cells were then washed in PBS and immediately imaged with confocal microscope (63× oil objective lens, Olympus).

For measurement of lysosomal acidification by flow cytometry, MDA‐MB‐231 and MCF‐7 cells were cultured in 6‐well plate for overnight and incubated for 24 hour in indicated experimental conditions. Cells were then stained with fluorescent LysoTracker Red DND‐99 probe as described above and thereafter analysed by FACS Calibur flow cytometer (Becton Dickinson).

### 2.11 In vivo experiments

Orthotopic 4T1 syngenic tumour model was used for this study. Prior approval on the study protocol was obtained from Institutional Animal Ethics Committee (IAEC) of the CSIR‐Central Drug Research Institute. Approximately 10^6^ cells (in 100 µL PBS) were transplanted into the mammary fat pad of 5‐ to 6‐week‐old female nude Crl: CD1‐Foxn1^nu^ mice. After 9 days and a tumour volume of approximately 50‐100 mm^3^, animals were randomly divided into two experimental (control and treatment) groups. TN‐16, dissolved in 100 µL of vehicle (5% PEG400 and 5% Tween 20 in PBS), was administered daily via an intraperitoneal injection at doses of 1 mg/kg body weight. Animals in control group received equal volume of the vehicle. Tumour size (length and width) was measured by vernier calipers on every other day after the start of treatment. The tumour volume (TV) was calculated using the formula TV = 0.5 × W^2^ × L.

### 2.12 Statistical analysis

Data are represented as mean value ± standard error (SE) of at least 3 independent experiments. Statistical analyses were performed by GraphPad Prism or Microsoft Excel using Student's *t* test. A *P* < .05 or less was considered as statistically significant.

## 3 RESULTS

### 3.1 TN‐16 inhibits cell proliferation by promoting G2/M arrest and induction of apoptosis

**Figure 1:**
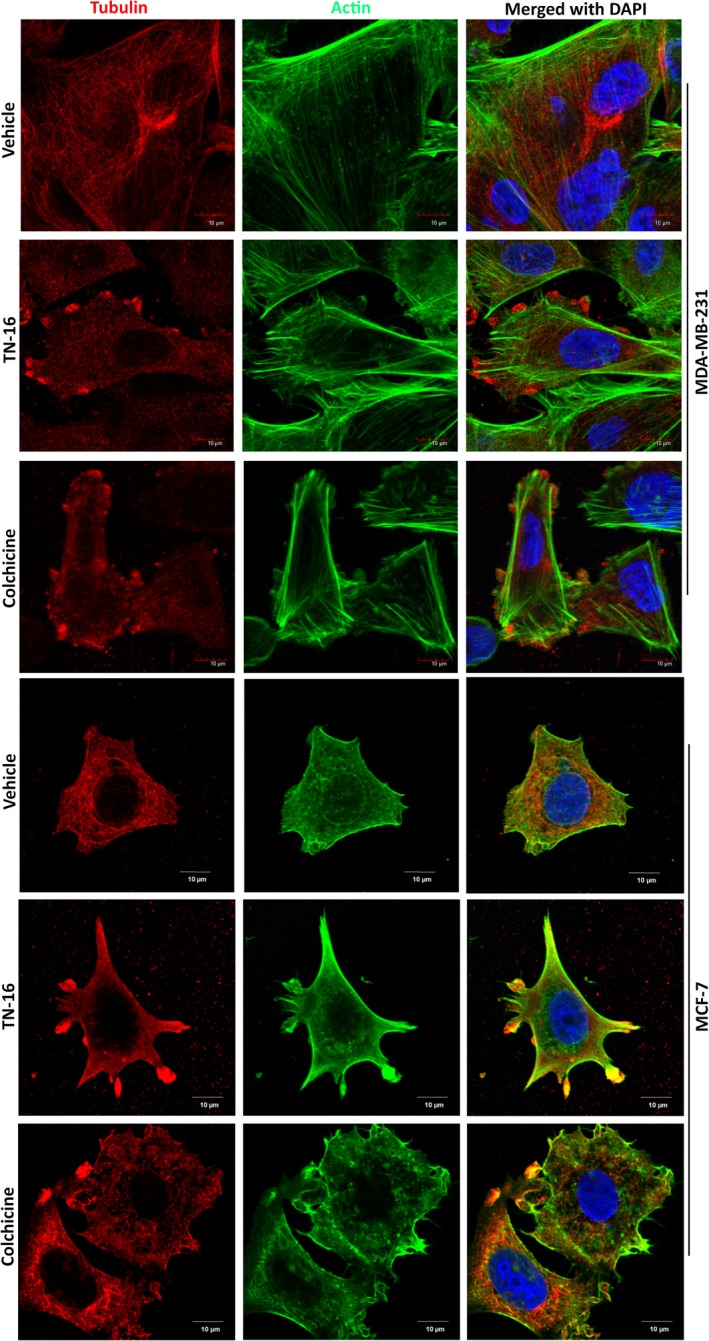
TN‐16 disrupts microtubular dynamics. MDA‐MB‐231 and MCF‐7 cells were treated with vehicle (DMSO) or TN‐16 (1.5 µmol/L) for 24 h. Cells were then probed with Alexa Fluor 488 phalloidin (for staining of actin filaments) and anti‐β‐tubulin antibody followed by Alexa Fluor 594‐conjugated secondary antibody (for staining of microtubules). Images were captured using confocal microscope at 63× magnification. Colchicine (1 µmol/L for 24 h) was used as positive control

**Figure 2:**
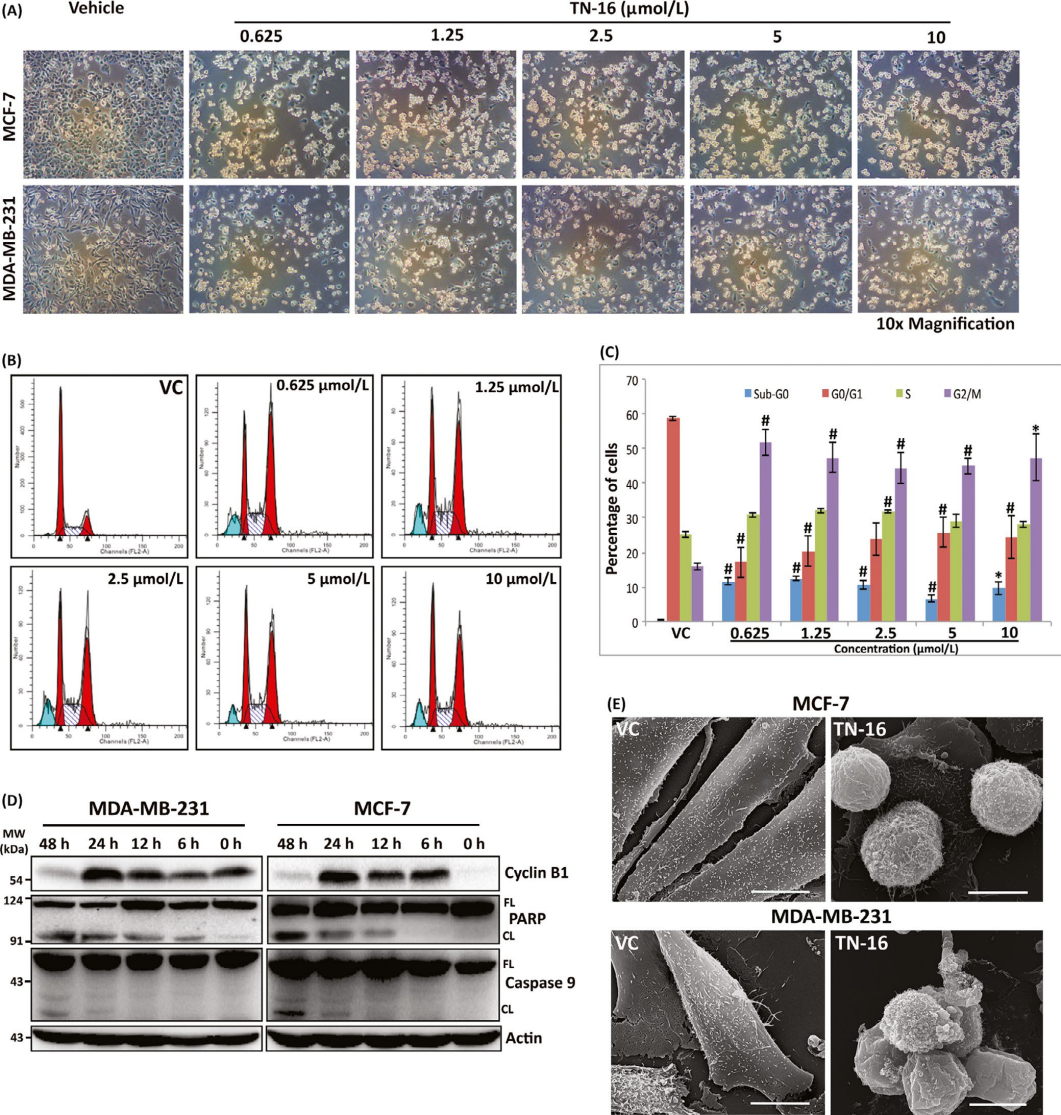
TN‐16 induces G2/M cell cycle arrest and apoptosis. A, Representative microscopic images of MCF‐7 and MDA‐MB‐231 cells after 24 h incubation with TN‐16 at indicated concentrations. B, MDA‐MB‐231 cells were treated with vehicle or TN‐16 at various concentrations for 24 h. The distribution of cell cycle was analysed by flow cytometry after propidium iodide staining of cellular DNA. C, Histogram showing distribution of cells at different phases of cell cycle before and after TN‐16 treatment. Data represent mean ± SE of three independent experiments. *P < .05, ^#^ *P* < .005 compared with control group. D, MCF‐7 and MDA‐MB‐231 cells were treated with 1.25 µmol/L TN‐16 for indicated time periods. The expression of cell cycle regulatory protein (cyclin B1) and biochemical markers of apoptosis (cleavage of PARP and Caspase‐9) were analysed by immunoblotting. E, SEM micrographs displaying surface ultrastructure of MDA‐MB‐231 and MCF‐7 cells before and after TN‐16 treatment (1.25 µmol/L for 24 h)

**Figure 3:**
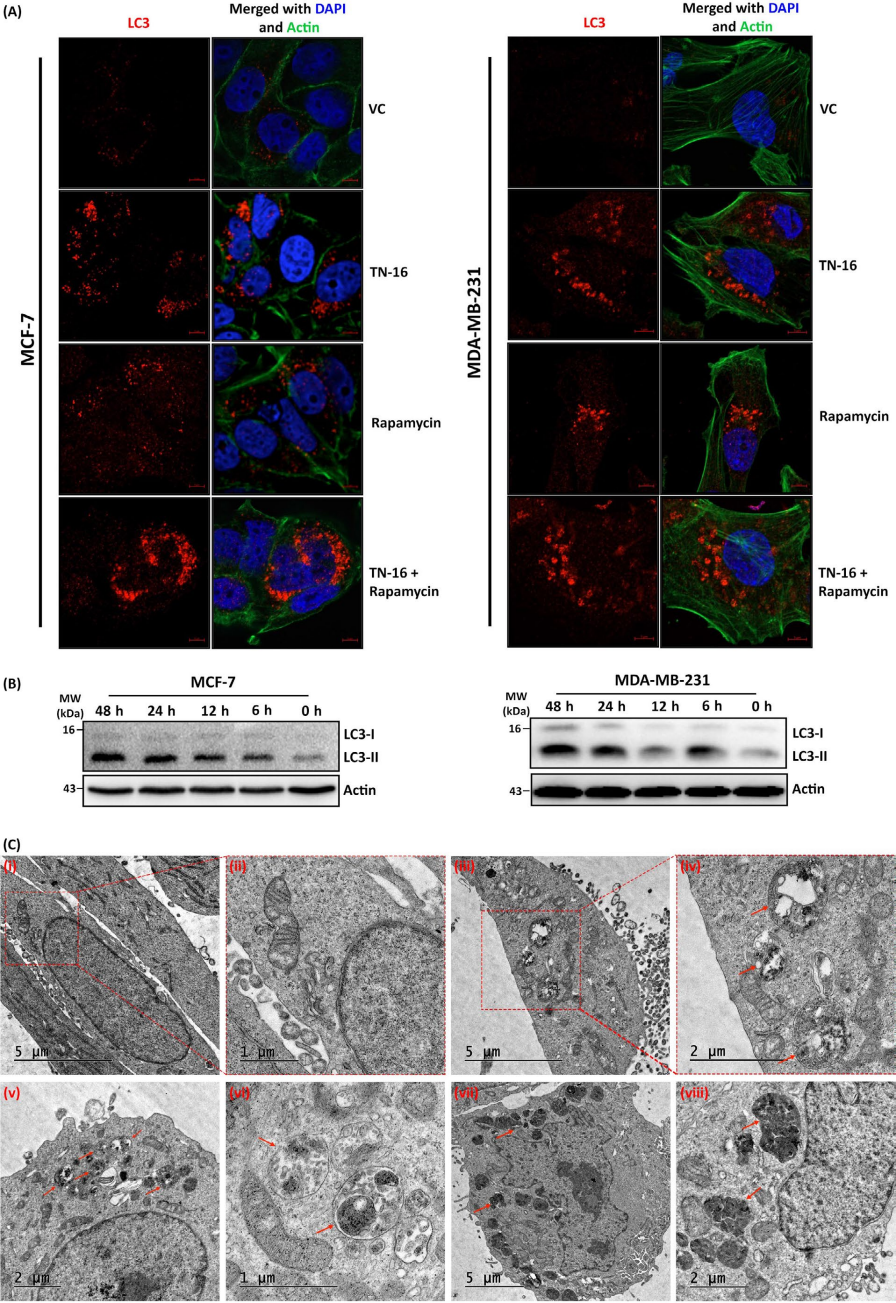
Morphological and biochemical characterization of TN‐16 induced autophagosome accumulation. A, Confocal micrographs of immunostained LC3 in MCF‐7 and MDA‐MB‐231 cells before and after TN‐16 treatment (1.25 µmol/L for 24 h). Actin was stained with Alexa Fluor 488 phalloidin, and DAPI was used for nuclear staining. Rapamycin (100 nmol/L for 24 h) was used as standard autophagy inducer. B, Lipidation of LC3 was assessed by immunoblotting after incubation of MCF‐7 and MDA‐MB‐231 cells with TN‐16 (1.25 µmol/L) for various time periods. C, Morphological characterization of TN‐16 induced autophagic flux in MDA‐MB‐231 cells using transmission electron microscopy. (C‐i to C‐ii) Control cells show typical morphological features of a healthy cell with normal mitochondria and intact nucleus. (C‐iii to C‐vi) Treated cells show increased number of autophagic vacuoles in MDA‐MB‐231 cells upon TN‐16 exposure (1.25 μmol/L, 24 h), while various degradative cellular components were observed in TN‐16–treated cells (C‐vii to C‐viii)

In our study, we first validated the microtubule destabilizing activity of TN‐16 in MCF‐7 and MDA‐MB‐231 breast cancer cells by confocal microscopy after treating them for 24 hours at 1.5 µmol/L concentration. Control cells showed well organized cytoskeletal dynamics with uniform array of actin and tubulin networks. On the contrary, treated cells displayed collapse of the microtubule network. This was associated with disruption of uniformity in distribution of actin filaments (Figure 1). Subsequently, we evaluated the efficacy of TN‐16 in inhibiting in vitro cell proliferation. Light‐microscopic observations of cells treated with TN‐16 at different concentrations for 24 hours revealed marked alterations in cellular morphology (as compared to untreated group) such as cell shrinkage and detachment from the surface that are indicative of growth inhibition (Figure 2A). We further explored effect of TN‐16 on cell cycle distribution. Flow cytometry analyses showed that TN‐16 treatment for 24 hours at various concentrations led to significant accumulation of cells at G2/M phase in comparison with the control groups (Figure 2B,C and Figure S1). The increase in G2/M phase cells by TN‐16 was associated with decreased G1 population and subtle upregulation of apoptotic cells in sub‐G0 phase in MDA‐MB‐231 cells. Similarly, there was marginal increase in percentage of cells at S phase after TN‐16 treatment. For successive in vitro experiments, cells were treated with 1.25 µmol/L of TN‐16. This selection was based on the observations of relatively more apoptotic population in 1.25 µmol/L treatment group (Figure 2C) and apparently similar morphological (altered) appearance in cells treated with TN‐16 at 1.25 µmol/L and above concentrations (Figure 2A). We next examined expression of cyclin B1 that plays a crucial role in cell cycle progression in mitotic phase. Incubation of cells with TN‐16 resulted in induction of cyclin B1 which reached a peak at 24 hours post‐incubation, but the level was markedly decreased thereafter at 48 hours post‐treatment (Figure 2D). Reduction of cyclin B1 at 48 hours post‐treatment is indicative of slippage of certain cell population from (tubulin‐binding agent‐induced) mitotic arrest which eventually become apoptotic or senescent or can regain proliferative activity with highly abnormal genome.^25^ In the following experiments, we investigated whether TN‐16–dependent cell growth inhibition is associated with apoptosis. MCF‐7 and MDA‐MB‐231 cells were treated with TN‐16 for various time points at indicated concentration. Protein lysates from vehicle and TN‐16–treated cells were examined for cleavage of PARP and caspase‐9 by Western blot assay. As shown in Figure 2D, TN‐16 induced marked fragmentation of PARP and caspase‐9 in both the cell lines suggesting induction of apoptosis by TN‐16. Scanning electron microscopy of TN‐16–treated cells also revealed characteristic morphological features of apoptosis such as shrinking of cytoplasm, microvilli disappearance/reduction and membrane blebbing (Figure 2E). Supporting this, ultrastructural study of TN‐16–treated cells (by TEM) revealed rounding of cells with blebs coming off the plasma membrane. The nucleus was also observed to be irregular in morphology (Figure 3C). Taken together, these results provide strong evidences that TN‐16 inhibits cancer cell growth by triggering apoptosis and G2/M phase arrest.

### 3.2 TN‐16 triggers intracellular autophagosomes formation

Given the essential role of microtubules in regulation of autophagosome dynamics,^26^ most if not all tubulin inhibitors modulate autophagic machinery. Since TN‐16 was originally developed as microtubule destabilizing agent, here we investigated alterations of a microtubule‐associated protein light chain 3 (LC3), which is an established biomarker for detection of autophagy, by biochemical and morphological assays. Lipidation of LC3, by conjugating with phosphatidylethanolamine during autophagy, allows it to be redistributed and associated with autophagosomes which gives a typical puncta appearance in fluorescence microscopy. In our study, we immunostained LC3 and monitored its redistribution from cytosol to autophagosomes by confocal microscopy. As can be seen in Figure 3A, marked increase in the number of LC3‐specific punctate dots was observed in TN‐16–treated cells in comparison with the untreated controls. Similar results were obtained in morphological analysis, by fluorescence microscopy, of TN‐16–treated MDA‐MB‐231 cells with stable expression of GFP‐LC3 (Figure S2). Consistent with the morphological observations by confocal microscopy, marked increase in endogenous LC3‐II turnover, which is indicative of autophagosome formation, was observed during analysis of protein lysates from TN‐16–treated cells by Western blot assay (Figure 3B). We further employed transmission electron microscopy (TEM) to determine whether intracellular autophagosome dynamics is indeed modulated in response to TN‐16 exposure. Results indicated that incubation of MDA‐MB‐231 cells with TN‐16 at 1.25 µmol/L concentration for 24 hours caused an increase in the number of autophagic vacuoles (Figure 3C) in various stages of maturation. However, the content of the degradative compartments appeared very electron dense suggesting that the ability to degrade cargo may not be affected.

### 3.3 TN‐16 inhibits autophagic flux and impairs lysosomal proteolysis

**Figure 4:**
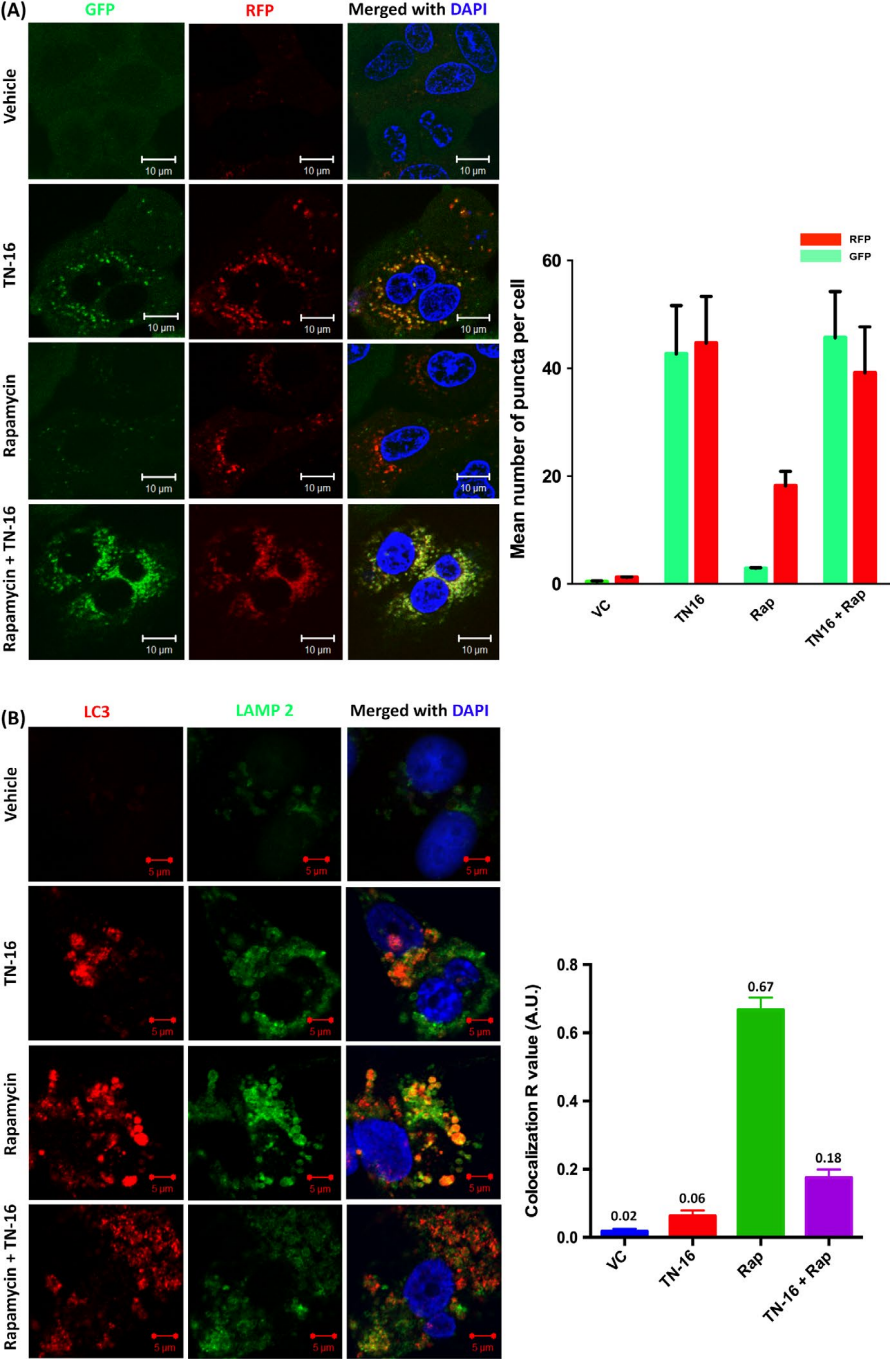
TN‐16 impairs autophagosome‐lysosome fusion. A, MDA‐MB‐231 cells, with stable expression of GFP‐LC3‐RFP‐LC3ΔG fluorescence probe, were treated with 1.25 µmol/L TN‐16 for 24 h. Representative confocal images (left panel) and bar graph (right panel) of mean puncta per cell (minimum 15 cells for each parameter) are shown. Rapamycin was used as standard autophagy inducer. B, Immunocytochemical analysis of LC3 and LAMP2 co‐localization in MDA‐MB‐231 cells before and after incubation with TN‐16 (left panel). Rapamycin was used as standard autophagy inducer. Co‐localization analysis (of cells in minimum 5 different fields in each experimental group) was performed using AIM 4.0 software where *R*, Pearson's correlation coefficient, was calculated and presented graphically (right panel)

Accumulation of autophagic vacuoles or LC3‐specific puncta or enhanced LC3‐II turn over does not necessarily mean increased autophagic process, because augmentation of cytosolic autophagosomes can occur either during induction of autophagy by upstream processes or inhibition of autophagolysosome formation at a later stage. To distinguish between these two mechanisms, we examined effect of TN‐16 on fusion of autophagosomes with lysosomes. To this end, we used a retroviral vector for stable expression of GFP‐LC3‐RFP‐LC3ΔG fluorescence probe in MDA‐MB‐231 cells. It is a dual fusion probe in which GFP‐LC3 is fused to the N terminus of conjugation‐deficient RFP‐LC3.^21^ During autophagy, autophagosome‐bound GFP‐LC3 is degraded (causing quenching of green fluorescence) in lysosome while conjugation‐deficient cytosolic RFP‐LC3ΔG serves as internal control with intact (red) fluorescence intensity, thereby resulting in decrease in GFP/RFP ratio. In agreement with this, we observed significant quenching of GFP fluorescence in cells treated with standard autophagy inducer rapamycin. On the contrary, marked increase in GFP‐LC3–specific puncta, as compared to control and rapamycin treatment group, was observed in TN‐16–treated cells (Figure 4A). In both the experimental groups (TN‐16 alone and rapamycin in combination with TN16), RFP fluorescence intensity was optimum and thereby suggesting diminished autophagolysosome formation upon TN‐16 treatment (Figure 4A). Additionally, we performed immunocytochemical analyses of MCF‐7 and MDA‐MB‐231 cells to authenticate distribution pattern of LC3 and LAMP2 upon TN‐16 treatment where we observed diffuse distribution of LC3 in untreated controls (Figure 4B and Figure S3). However, rapamycin treatment caused ample accumulation of LC3‐specific puncta; most of which were co‐localized with LAMP2 (*R* = .67 ± .04) indicating maturation of autophagolysosomes (co‐localization analysis was performed using AIM 4.0 software where *R*, Pearson's correlation coefficient, was calculated). In contrast, cells treated with TN‐16 alone or in combination with rapamycin showed much less accumulation (*R* = .06 ± .02 and .18 ± .02, respectively) of LC3 with LAMP2 (Figure 4B and Figure S3) and thus confirming TN‐16–mediated inhibition of autophagosome and lysosome fusion.

**Figure 5:**
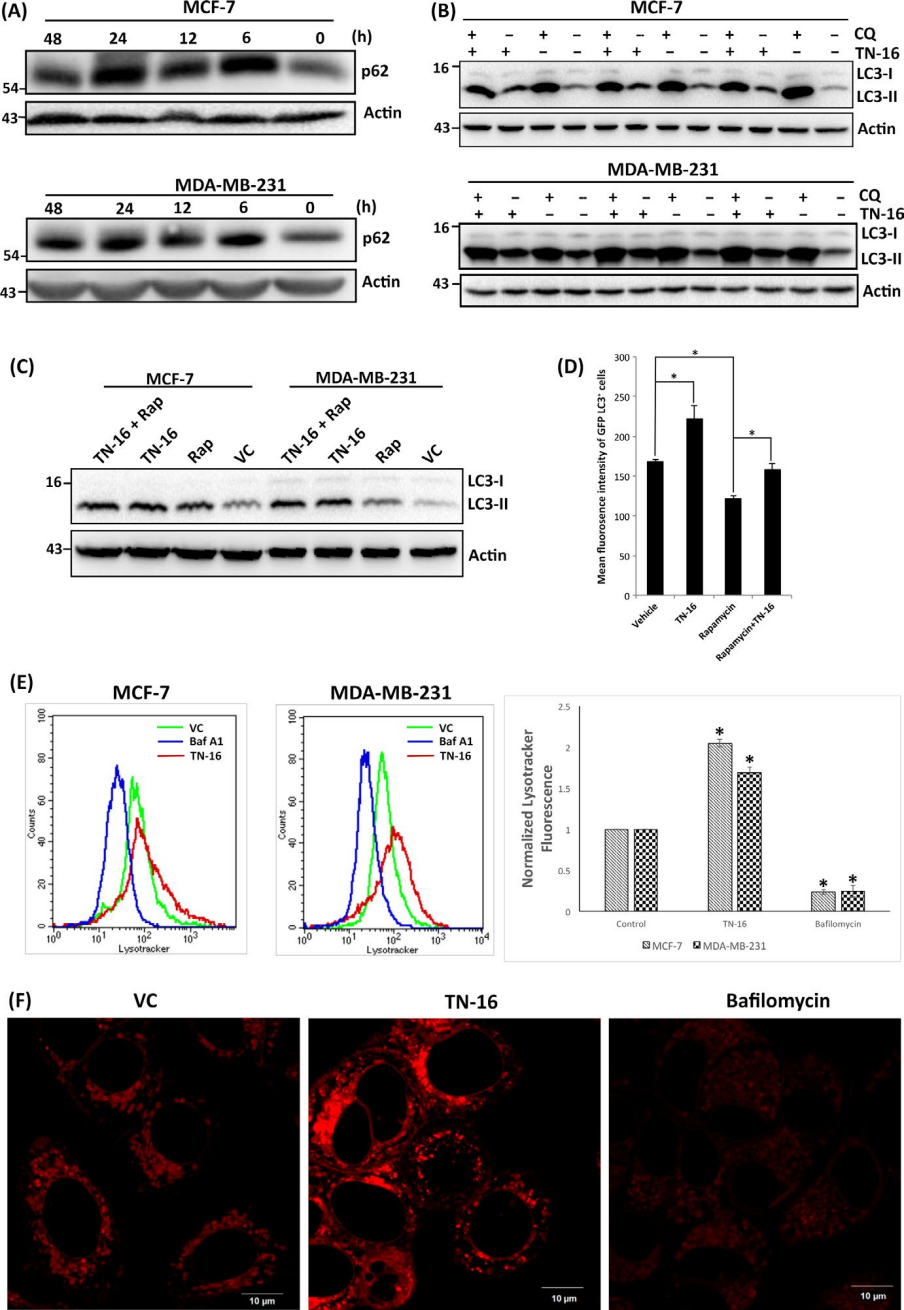
TN‐16 inhibits autophagic flux. A, Protein level of p62 in MCF‐7 and MDA‐MB‐231 cells was determined by immunoblot analysis after incubation with 1.25 µmol/L TN‐16 for indicated time points. B, TN‐16 (1.25 µmol/L for 24 h) mediated LC3 turn over examined by immunoblotting in the presence or absence of CQ (2 µmol/L). C, Effect of TN‐16 on rapamycin‐mediated LC3 lipidation was investigated by Western blot assay. D, MDA‐MB‐231 cells, stably expressing GFP‐tagged LC3, were treated with either TN‐16 (1.25 µmol/L), rapamycin (100 nmol/L) or both for 24 h. Relative fluorescence intensity of GFP‐LC3 was measured by flow cytometry. Data represent mean ± SE of three independent experiments. *P < .05. E, MCF‐7 and MDA‐MB‐231 cells, stained with LysoTracker^®^ red, were analysed by flow cytometry in indicated experimental conditions. Fluorescence histogram (left panel) and normalized mean fluorescence intensity (with control condition) of three independent experiments (right panel) are shown. (*P < .005 compared to respective control group) F, Representative confocal images of MDA‐MB‐231 cells after staining with LysoTracker^®^ red

In autophagy, p62 acts as an adaptor molecule for recruitment of ubiquitylated substrates into nascent autophagosomes through tethering with the membrane‐bound LC3‐II and subsequently degraded in autophagolysosomes together with cargo.^27^, ^28^ Therefore, reduction of p62 level is widely used as an indicator for autophagic flux. In our study, we assessed p62 expression in breast cancer cells before and after incubation with TN‐16 to further confirm its effect on autophagic process. As shown in Figure 5A, incubation of cells with TN‐16 resulted in marked increase in p62 expression suggesting impaired degradation autophagic cargo. To further validate our observations on TN‐16–dependent inhibition of autophagic flux, we carried out immunoblot analysis to assess LC3‐II turnover by TN‐16 in the presence or absence of lysosomotropic agent chloroquine (CQ) that enhances intralysosomal pH to interfere substrate degradation. Figure 5B shows that combined treatment of both TN‐16 and CQ did not have any significant additive effect and thus corroborating with the morphological data suggesting that TN‐16 is indeed an inhibitor of autophagic flux. We further investigated effect of TN‐16 on rapamycin‐mediated LC3‐II turnover. Here, we observed additionally increased LC3‐II level in cells that are co‐incubated with rapamycin and TN‐16 in comparison with the rapamycin or TN‐16 alone (Figure 5C) and thereby validating the inhibitory role of TN‐16 on autophagic flux. To add further, we analysed MDA‐MB‐231 cells stably expressing GLP‐LC3 by flow cytometry at different experimental conditions. Cells treated with rapamycin for 24 hours showed significant decrease in average fluorescence intensity as compared to the vehicle‐treated controls (Figure 5D) due to lysosomal degradation (quenching) of GFP‐LC3. On the contrary, incubation of cells with TN‐16 alone or in combination with rapamycin prevented reduction of fluorescence signal and thereby validating impaired autophagolysosomal degradation of substrates in TN‐16–treated cells.

Having established the inhibitory role of TN‐16 in autophagic flux, we further asked whether it works similar to lysosomal inhibitors. To this end, vacuolar acidification was measured by confocal microscopy and flow cytometry after staining the cells with a fluorescent acidotropic probe, LysoTracker^®^ red. In both assays, we found a dramatic increase of fluorescence intensity in TN‐16–treated cells as compared to the untreated control whereas the intensity was much lower upon incubation of the cells with lysosomal inhibitor bafilomycin (Figure 5E,F). Thus, our results indicate that the mechanism of autophagic flux inhibition by TN‐16 is distinct from the effect of lysosomal inhibitors. Increased acidification in TN‐16–treated cells is probably due to enhanced lysosomal volume, because of microtubule destabilization, instead of induction of autophagolysosmes.^29^

### 3.4 Interplay between TN‐16–mediated inhibition of autophagic flux and induction of apoptosis in human breast cancer cell death

**Figure 6:**
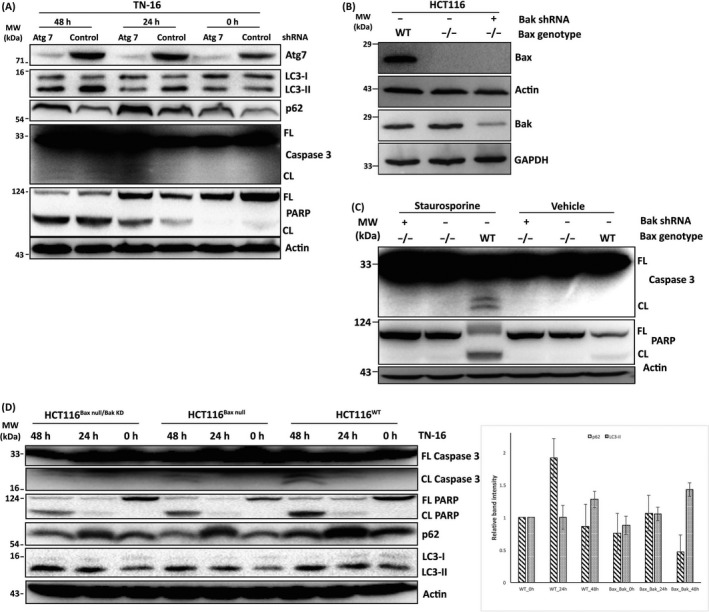
Cross‐regulation between TN‐16–mediated induction of apoptosis and impaired autophagic flux. A, HCT116 (Bax+/‐) cells were transduced with lentiviral vectors for stable silencing of Atg7. The cells were then incubated with TN‐16 (1.25 µmol/L) for different time points. Cell lysates were subsequently probed with indicated antibodies. B, The absence of Bax and reduced expression of Bak in experimental cell lines was validated by Western blot assay. C, HCT116^WT^ and isogenic Bax^null^ and Bax^null^/Bak^KD^ cells were treated with staurosporine (200 nmol/L for 24 h) and analysed by Western blot assay for apoptotic markers D, TN‐16 (1.25 µmol/L for 24 and 48 h)‐treated HCT116^WT^, Bax^null^ and Bax^null^/Bak^KD^ cells were subjected to immunoblot assay to determine expression/activation various biochemical markers of apoptosis and autophagy (left panel). Densitometric quantification of LC3‐II turnover and p62 expression (n = 3) is shown in bar graph (right panel)

Concurrent inhibition of autophagic flux and activation of apoptosis by TN‐16 in breast cancer cell lines raised the question whether these two events are interlinked or they occur independently. To address this, we inhibited autophagy in HCT116 cells by shRNA‐mediated downregulation of *atg7* gene which plays essential role in autophagosome formation. The knockdown efficiency of shRNA was confirmed by Western blot assay showing marked suppression in Atg7 expression (Figure 6A). In agreement with previous reports,^30^, ^31^ Atg7 downregulation was associated with reduced conversion of LC3‐I to LC3‐II and accumulation of p62 (Figure 6A) suggesting deficiency in autophagy. We observed that suppression of autophagy by shRNA‐mediated silencing of Atg7 led to an increase in TN‐16–induced apoptosis. This was evident as enhanced fragmentation of PARP and activation (cleavage) of caspase‐3 in Atg7 knockdown cells in comparison with the autophagy‐proficient cells expressing scrambled shRNA sequence (Figure 6A).

To further determine how pro‐apoptotic activity of TN‐16 influences its autophagic flux inhibitory effect, we blocked apoptosis by shRNA‐mediated downregulation of Bak in Bax‐deficient (Bax^null^) HCT116 cells. Impaired expression of Bax and Bak in test cell lines was confirmed by immunoblotting (Figure 6B). Next, we treated these cells with standard apoptosis inducer staurosporine (STS) at 200 nmol/L concentration for 24 hours and compared expression of different biochemical markers of apoptosis with wild‐type control cells. Here we observed significant reduction of STS‐induced apoptosis in cells that are either deficient in Bax (Bax^null^) alone or with simultaneous depletion of Bax and Bak (Bax^null^/Bak^KD^). The effect was evident as decrease/absence of PARP and caspase‐3 cleavage after STS treatment (Figure 6C). In the following experiments, cells were incubated with TN‐16 for different time points and Western blot assay was performed to analyse protein lysates for various apoptosis and autophagy markers. Similar to the results obtained in STS‐treated cells, TN‐16–induced cleavage of PARP and caspase‐3 was markedly decreased in Bax^null^ and Bax^null^/Bak^KD^ cells (Figure 6D) and thus validating impaired apoptosis. Analyses of HCT116 cell lysates by immunoblotting also revealed induction of LC3‐II turnover and accumulation of p62 protein by TN‐16 (Figure 6D) which is in agreement with our earlier findings in human breast cancer cell lines suggesting blockade of autophagic flux. Conversion of LC3‐I to LC3‐II was further enhanced in cells with reduced level of Bax and Bak (Figure 6D). On the contrary, we observed decrease in TN‐16–mediated accumulation of p62 in Bax^null^ and Bak^KD^ cells in comparison with their isogenic wild‐type controls (Figure 6D), indicating partial relieve of TN‐16–induced autophagic flux blockade.

### 3.5 TN‐16 inhibits in vivo growth of orthotopic mouse model of breast cancer

**Figure 7:**
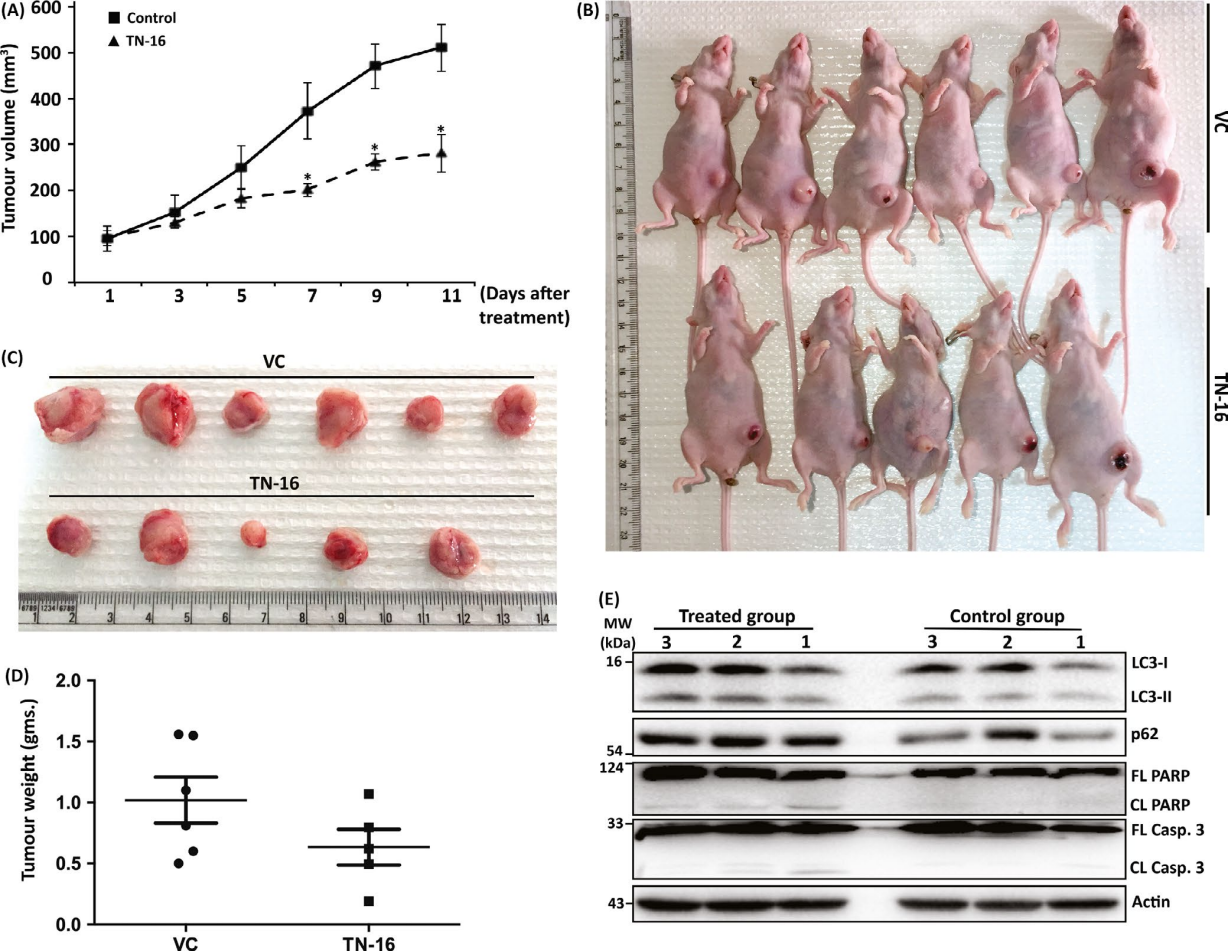
TN‐16 inhibits murine breast tumour growth in vivo. The 4T1 mouse mammary tumour cells were inoculated into the mammary fat pad of female nude mice. Once the tumour reached approximately 100 mm^3^ in size, the animals were treated either with vehicle or TN‐16 (1 mg/kg body weight) by i.p. daily for 11 days. A, Tumour volumes were calculated by measuring length and width on alternate days. Data, comparing vehicle versus TN‐16–treated group, were analysed by two tail *t* test for each denoted day after treatment. *P < .05 compared with control group. Gross appearance of tumour bearing mice (B) and harvested tumours (C) at the end of the study. D, Average weight of tumours from mice in each group. E, Western blot analysis of tumour lysates to determine expression of indicated proteins

In the present study, 4T1 cells were implanted into the mammary fat pad of nude mice to induce orthotropic model of breast cancer. By day 9, a palpable mass of tumour was developed measuring approximately 100 mm^3^ volume. The mice were then treated at every alternate day with either TN‐16 (@ 1 mg/kg B.W) or vehicle (5% PEG400 + 5% Tritonx100 in PBS) through intraperitoneal injection for 11 days. As shown in the Figure 7A‐C, TN‐16 treatment led to the significant reduction in tumour growth in comparison with the control. Likewise, average weight of the harvested tumours in treatment group was much less compared with the control (Figure 7D).

Finally, we analysed tumour tissue‐derived protein lysates by immunoblotting to determine expression level of various apoptosis and autophagy markers. As can be seen in Figure 7E, level of cleaved PARP and caspase‐3 is increased in tumours isolated from treated mice and thus validating contribution of apoptosis in TN‐16–mediated tumour growth inhibition. Consistent with results of our in vitro studies, biochemical analysis of tumour tissue lysates revealed blockade of autophagic flux by TN‐16 which was evident as simultaneous accumulation of p62 and increased turnover of LC3‐II in protein lysates obtained from treatment group (Figure 7E). Altogether, these results indicate that suppression of tumour growth by TN‐16 is associated with impaired autophagic flux and apoptosis.

## 4 DISCUSSION

Promise of tubulin‐binding agents in treatment of cancer has been the main focus in past decades. Due to their essential role in several key cellular processes such as cell division, motility and intracellular trafficking, microtubules represent an attractive target for anti‐cancer drug discovery. Nevertheless, recent developments on the understanding on importance of microtubules in autophagic process^11^, ^32^ have thrown insights into the possibilities that their anti‐cancer efficacy may be partially attributable to their ability to modulate autophagy. In general, small molecules that destabilize microtubules disrupt autophagic flux and thereby cause accumulation of toxic protein aggregates and/or damaged organelles which in turn promote cancer cell death.^33^ In this study, we investigated efficacy of TN‐16, a colchicine site binding agent, for anti‐cancer activity and its underlying mechanisms. We found that TN‐16 induces apoptosis in human cancer cell lines which is in agreement with earlier observations.^34^ We also demonstrated TN‐16–mediated inhibition of in vivo tumour growth and blockade of late‐stage autophagy which further facilitates its pro‐apoptotic activity.

As mentioned above, dynamics of autophagosome formation as well as its ensuing maturation essentially rely on the integrity of cytoskeletal network and especially on microtubule assemblage.^35^ TN‐16 was synthesized as tubulin targeting agent which interferes microtubule assembly through reversible binding to the colchicine‐sensitive site of tubulin.^14^ Thus, it is quite likely that TN‐16, by virtue of its ability to destabilize tubulin network, may modulate autophagy. Keeping this in mind, we examined various biochemical and morphological characteristics of autophagy in TN‐16–treated cells. Our data revealed that TN‐16 triggered increased turnover of LC3‐II both in vitro and in vivo and notable accumulation of LC3‐specific puncta in cell cytoplasm suggesting induction of autophagosomes which can occur either due to activation of autophagic flux or because of inhibition of autophagosome and lysosome fusion. In the following experiments, we used different approaches to distinguish between maturation of autophagolysosomes and defects in autophagic flux, both of which essentially triggers autophagic vacuoles in association with increased conversion of LC3‐I to LC3‐II. Here, we observed accumulation autophagy‐specific substrate p62 upon TN‐16 treatment. In line with this finding, incubation of cells with TN‐16 in combination with lysosomotropic agent (CQ) failed to produce any additive effect in LC3‐II turnover suggesting interference of autophagic flux. Microtubules (MT) influence autophagic process in different ways. LC3 and other crucial effector molecules that are engaged in the early steps of autophagosome formation have been shown to directly or indirectly interact with MTs,^26^, ^36^, ^37^ indicating an intricate role of MTs in assembly of autophagosomal structures. On the other hand, several reports demonstrated crucial role of MT for trafficking of autophagosomes towards lysosomes^38^ and subsequent maturation of autophagolysosomes by facilitating juxta‐nuclear positioning of these vesicles.^26^ In our study, immunocytochemical staining for LC3 and LAMP2 revealed lack of lysosome fusion with autophagosomes. This finding was further validated by confocal microscopy using a recombinant GFP‐LC3‐RFP‐LC3ΔG fluorescence probe where reduced quenching of autophagosome‐bound GFP‐LC3 was observed in TN‐16–treated cells because of diminished autophagosome‐lysosome fusion. Altogether, above results suggest that TN‐16–dependent impaired autophagic flux is probably due to defective autophagosome trafficking along the disrupted MTs. Nonetheless, TEM analysis of TN‐16–treated cells showed electron‐dense cellular degradative compartments (DGCs) suggesting no change in the ability of autophagic cargo degradation by the selective compartments. On the contrary, detailed investigation of various markers/steps in the autophagic process, by established technics, revealed that the molecule prevents fusion of autophagosomes with lysosome. The presence of electron‐dense degradative compartments in our TEM data can be explained by the fact that the observed outcome may be due to the cellular stress induced by TN16 because of other pronounce cellular alterations which may indirectly contribute in partial induction of autophagic flux. It is interesting to note that our results are in agreement with similar observations reported recently for standard autophagosome‐lysosome fusion inhibitor CQ, where it has been shown that CQ inhibits autophagy by blocking autophagosome‐lysosome fusion without affecting the degradation capacity of lysosomes.^39^

A complex relationship exists between autophagy and apoptosis. These two well‐controlled biological processes play vital roles in development and homeostasis of multicellular organisms. Autophagy and apoptosis can occur concurrently in the same cell and may influence each other either positively^40^ or negatively^41^ depending upon cell type, cellular metabolic homeostasis, availability of extracellular nutrient and triggering stimuli.^42^ In the present study, the fact that TN‐16 treatment led to a steady increase in the expression of biochemical markers of apoptosis in association with accumulation of p62 (adaptor protein for autophagy substrates) supports the notion that inhibition of autophagy lead to apoptosis.^43^ Blockade of autophagy either at early stage or late stage can trigger apoptosis. Results from our study indicated late‐stage inhibition of autophagic process by TN‐16. Accumulation of misfolded proteins and damaged mitochondria due to impaired autophagic flux may contribute in induction of apoptotic cell death^44^ by TN‐16. It is also quite likely that bioenergetics shortage because of TN‐16–dependent blockade of autophagic flux resulted in activation of apoptosis.^43^ We further knocked down ATG7 to investigate how early‐stage inhibition of autophagy affect TN‐16–induced cancer cell death. Autophagy suppression by depletion of ATG7 resulted in further increase in apoptosis. The observed outcome indicates an additive (late as well as early) effect of autophagy inhibition on TN‐16–dependent activation of apoptosis. We next sought to determine whether TN‐16–induced apoptosis has any impact on its autophagy modulatory activity. Here, we used HCT116 cell line which exhibits reduced apoptotic response due to depletion of essential proteins (Bax and Bak) of apoptotic machinery. The apoptotic resistant cells displayed more LC3‐II turnover and relatively reduced p62 accumulation, upon TN‐16 treatment, than wild‐type control cells suggesting derepression of TN‐16–mediated autophagic flux blockade. Our results corroborate previous findings where genetic deletion of Bax and Bak resulted in massive induction of autophagy to compensate impaired apoptosis.^45^, ^46^ Collectively, our results demonstrate that autophagic flux inhibition by TN‐16 promoted apoptotic cell death.

Summing up, this study demonstrates that TN‐16 inhibits in vitro and in vivo cancer cell growth through blockade of autophagic flux with simultaneous induction of apoptosis. As a MT destabilizing agent, TN‐16 prevented trafficking of autophagosomes along the tubulin track and thereby inhibited autophagosome‐lysosome fusion at later stage of autophagic process. We also found that TN‐16–induced cancer cell death is mediated in part by activation of apoptosis and blockade of autophagic flux facilitated its pro‐apoptotic activity. Taken together, this study concludes that TN‐16 can be a potential autophagy inhibitor which can be used alone or in combination with standard therapeutic agents to induce cancer cell death.

## CONFLICT OF INTEREST

The authors declare no conflict of interest.

## AUTHOR CONTRIBUTIONS

MH, RS, PP, MM, KC and DG performed most of the laboratory experiments. KM and KS contributed in confocal microscopy and image analysis. AS and DD contributed in animal experiments. JS formulated and supervised the study. JS wrote the manuscript, and KM and DD edited the draft.

## Data Availability

The data that support the findings of this study are available from the corresponding author upon reasonable request.
