# Supplementary figures and images for: Microtubule disrupting agent‐mediated inhibition of cancer cell growth is associated with blockade of autophagic flux and simultaneous induction of apoptosis
Source: Cell Prolif. 2020 Mar 13;53(4):e12749. doi: 10.1111/cpr.12749 (PMC7162801; doi:10.1111/cpr.12749)

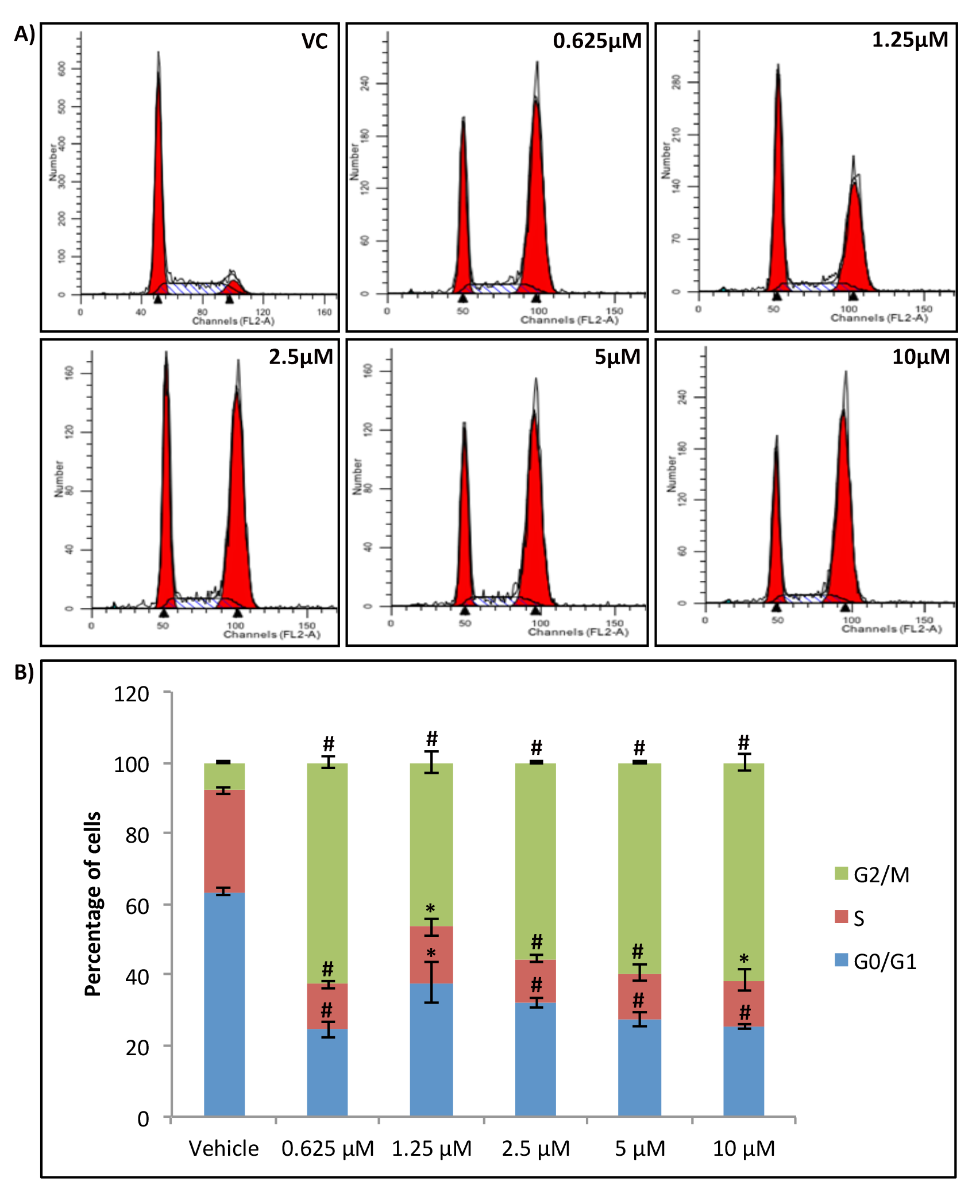

Supplement: Supplementary file 1 [file CPR-53-e12749-s001.tif]

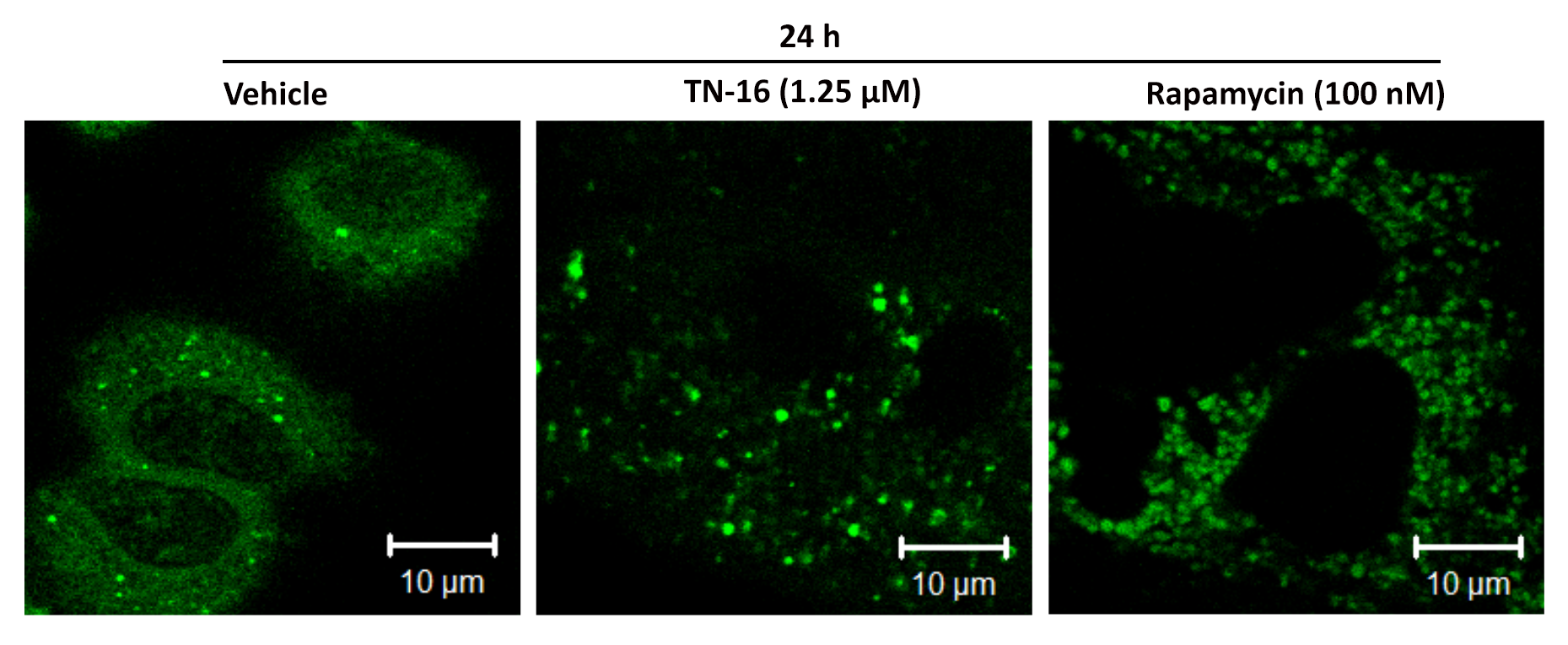

Supplement: Supplementary file 2 [file CPR-53-e12749-s002.tif]

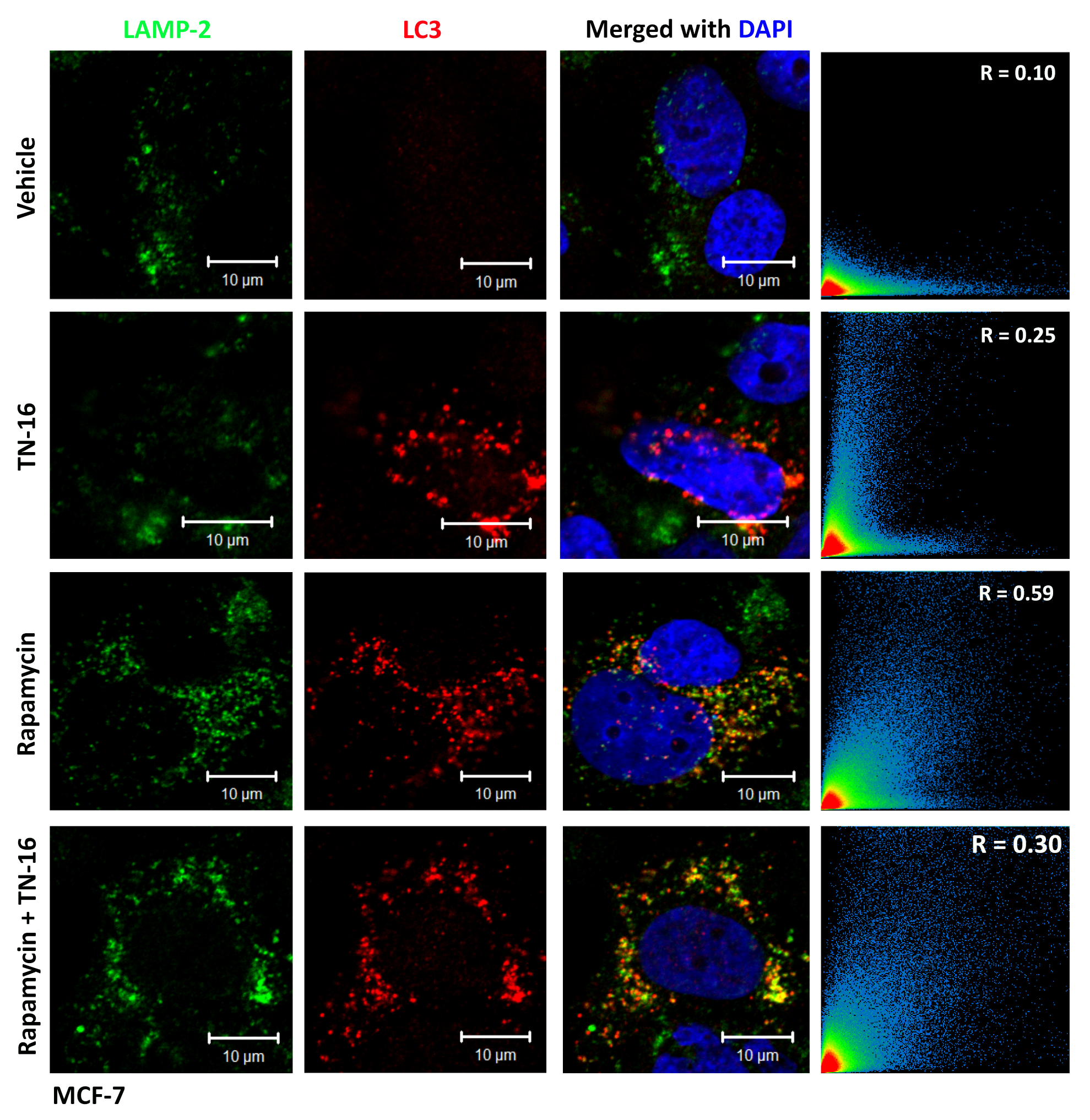

Supplement: Supplementary file 3 [file CPR-53-e12749-s003.tif]
