# Supplementary material for: Microtubule disrupting agent‐mediated inhibition of cancer cell growth is associated with blockade of autophagic flux and simultaneous induction of apoptosis
Source: Cell Prolif. 2020 Mar 13;53(4):e12749. doi: 10.1111/cpr.12749 (PMC7162801; doi:10.1111/cpr.12749)
Supplement: Supplementary file 4 [file CPR-53-e12749-s004.docx]

**Supplementary figure 1. TN-16 affects cell cycle in MCF-7 cells.** (a) MCF-7 cells were stained with propidium iodide after incubation with TN-16 at indicated concentrations for 24 h and analysed by flow cytometry for determination of cell cycle stage. (b) Percentages of cells at different phases of cell cycle before and after TN-16 treatment are represented graphically. **P* < 0.05, ^#^ *P* < 0.005 compared with control group.

**Supplementary figure 2. Effect of TN-16 on LC3 punctation.** MDA-MB-231 cells that are stably transduced by GFP-LC3 encoding retroviral vector were treated with TN-16 (1.25 µM) for 24 h. Distribution of LC3 specific puncta in presence and absence of TN-16 was determined by confocal microscopy.

**Supplementary figure 3.** Determining autophagic flux in TN-16 treated MCF-7 cells. Representative confocal micrographs of vehicle and TN-16 (1.25 µM for 24 h) treated MCF-7 cells after immunostaining with LC3 and LAMP2.
